# Novel initiator caspase reporters uncover previously unknown features of caspase-activating cells

**DOI:** 10.1242/dev.170811

**Published:** 2018-12-04

**Authors:** Luis Alberto Baena-Lopez, Lewis Arthurton, Marcus Bischoff, Jean-Paul Vincent, Cyrille Alexandre, Reuben McGregor

**Affiliations:** 1 Sir William Dunn School of Pathology, University of Oxford, South Parks Road, Oxfordshire, OX1 3RE, UK; 2 Biomolecular Sciences Research Complex, University of St Andrews, North Haugh, St Andrews, Fife, Scotland, KY16 9ST, UK; 3 The Francis Crick Institute, Midland Road, London, NW1 1AT, UK; 4 Faculty of Medical and Health Sciences, Molecular Medicine & Pathology, The University of Auckland, M&HS Building 502, 85 Park Road, Grafton, Auckland 1023, New Zealand

**Keywords:** Apical caspase reporter, Apoptotic nuclear migration, Historical caspase reporter, Interkinetic nuclear migration, Non-apoptotic caspase functions

## Abstract

The caspase-mediated regulation of many cellular processes, including apoptosis, justifies the substantial interest in understanding all of the biological features of these enzymes. To complement functional assays, it is crucial to identify caspase-activating cells in live tissues. Our work describes novel initiator caspase reporters that, for the first time, provide direct information concerning the initial steps of the caspase activation cascade in *Drosophila* tissues. One of our caspase sensors capitalises on the rapid subcellular localisation change of a fluorescent marker to uncover novel cellular apoptotic events relating to the actin-mediated positioning of the nucleus before cell delamination. The other construct benefits from caspase-induced nuclear translocation of a QF transcription factor. This feature enables the genetic manipulation of caspase-activating cells and reveals the spatiotemporal patterns of initiator caspase activity. Collectively, our sensors offer experimental opportunities not available by using previous reporters and have proven useful to illuminate previously unknown aspects of caspase-dependent processes in apoptotic and non-apoptotic cellular scenarios.

## Abstract (precis)

**Summary:** Novel initiator caspase sensors reveal previously unknown stages of apoptosis and stereotyped patterns of non-apoptotic caspase activation in physiological conditions.

## INTRODUCTION

The cysteine-dependent aspartate proteases, commonly known as caspases, are the major regulators of apoptosis, but also decisively modulate other essential biological functions independent of apoptosis (e.g. cell proliferation, cell differentiation and cell migration) (Aram et al., 2017; Baena-Lopez et al., 2017; Burgon and Megeney, 2017; Ellis and Horvitz, 1986; Fogarty and Bergmann, 2017; Hollville and Deshmukh, 2017; McIlwain et al., 2013; Miura, 2012; Mukherjee and Williams, 2017; Perez-Garijo, 2017; Songane et al., 2018). Accordingly, caspase malfunction in either apoptotic or non-apoptotic cellular scenarios often leads to disease initiation and progression (Aram et al., 2017; Baena-Lopez et al., 2017; Burgon and Megeney, 2017; McIlwain et al., 2013; Miura, 2012; Mukherjee and Williams, 2017; Perez-Garijo, 2017). As a first step towards characterising all of the caspase-related biological functions within complex cellular assemblies, it is important to devise methods that efficiently identify caspase-activating cells in live tissues. However, the repertoire of *Drosophila* caspase sensors with such properties is currently limited (Bardet et al., 2008; Ding et al., 2016; Florentin and Arama, 2012; Mazzalupo and Cooley, 2006; Schott et al., 2017; Takemoto et al., 2003; Tang et al., 2015; To et al., 2015; Xu et al., 2018).

Apoptotic caspases have been grossly classified as either initiator or executioner/effector depending on their early or late activation during the apoptosis (Baena-Lopez et al., 2017; Ramirez and Salvesen, 2018). The majority of genetically encoded caspase sensors described in *Drosophila* are based on short caspase-cleavage sites (DEVD or DQVD), recognised by effector caspases (Bardet et al., 2008; Ding et al., 2016; Florentin and Arama, 2012; Schott et al., 2017; Takemoto et al., 2003; Tang et al., 2015; To et al., 2015; Xu et al., 2018). One published sensor could potentially detect the activity of the initiator caspase 8 but this never been validated in *Drosophila* somatic tissues (Mazzalupo and Cooley, 2006). In order to make these reporters compatible with live-imaging techniques, they have often incorporated different fluorescent proteins at both ends of the caspase-recognition site (Bardet et al., 2008; Florentin and Arama, 2012; Mazzalupo and Cooley, 2006; Schott et al., 2017; Takemoto et al., 2003; To et al., 2015; Xu et al., 2018). Some of these sensors have exploited changes in the subcellular localisation of fluorescent proteins to visualise caspase activation (Bardet et al., 2008), whereas others have relied on split fluorophores that only shine after caspase-mediated cleavage (Mazzalupo and Cooley, 2006; Schott et al., 2017; To et al., 2015). Although these methods are undoubtedly powerful, even in non-lethal scenarios (Kanuka et al., 2005), they share some limitations. They cannot provide a temporal perspective of caspase activation over long periods of time and they do not enable straightforward genetic manipulation of caspase-activating cells. Moreover, their activation requires the enzymatic activity of effector caspases, and therefore they are not functional in biological contexts without the participation of the entire caspase pathway, a situation frequently observed in non-apoptotic scenarios (Kondo et al., 2006; Napoletano et al., 2017; Ouyang et al., 2011; Wells et al., 2006). Some of these issues have been partially overcome by two recent constructs that have incorporated a CD8 membrane retention domain and a transcriptional activator (Gal4) flanking the caspase-cleavage motif (Ding et al., 2016; Tang et al., 2015). However, these reporters still rely on an effector caspase cleavage motif (DQVD), and the inclusion of a Gal4 fragment impedes their usage in combination with pre-existing Gal4 lines.

Here, we describe a new set of highly sensitive caspase reporters that overcome all the aforementioned shortcomings by incorporating an enzymatically dead but still cleavable template of the effector caspase Drice. This configuration ensures direct excision by initiator caspases, while preventing their ability to trigger apoptosis. Our reporters also include additional features that have proven useful in unearthing new nuclear movements in pre-apoptotic cells as well as previously unknown biological properties of caspase-activating cells in different *Drosophila* tissues.

## RESULTS

### Rational design of a novel Drice-based sensor (DBS)

**Fig. 1.**
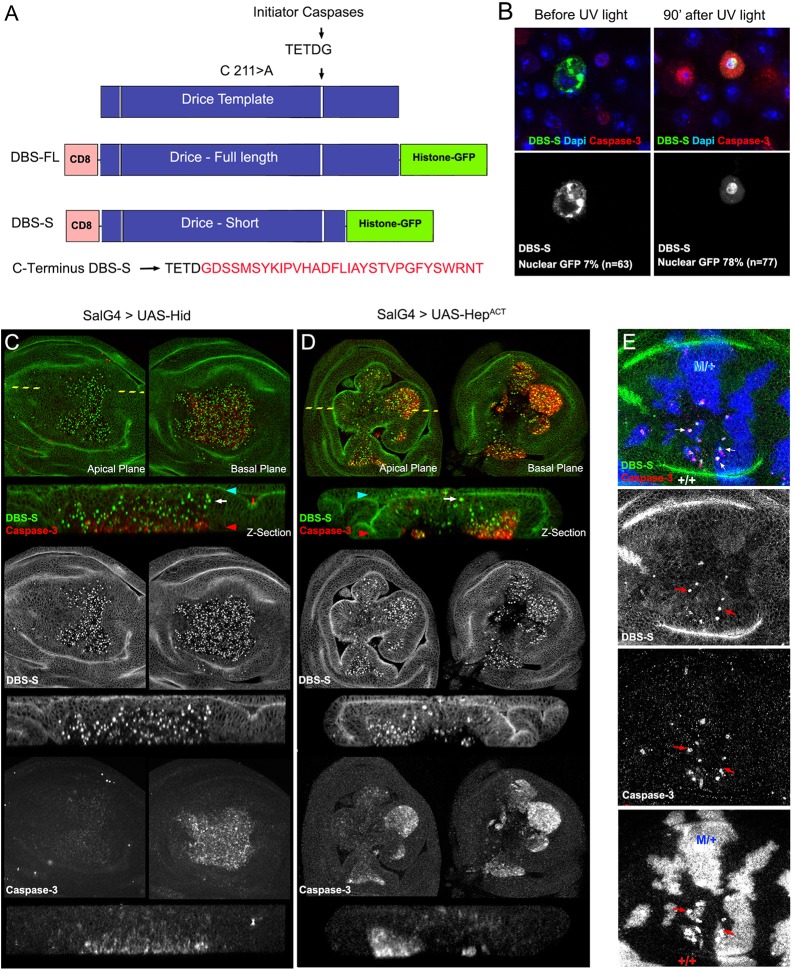
**Rational design and validation of the Drice-based sensor (DBS-S).** (A) Schematic of the Drice template indicating the mutation introduced in the catalytic residue (C211>A; cysteine-to-alanine substitution) and the initiator caspase cleavage site (TETD/G). Rational design of constructs generated from the Drice template [Drice-based sensor with full-length histone GFP (DBS-FL) and Drice-based sensor with short histone GFP (DBS-S)]. Amino acid sequence kept in DBS-S after the initiator cleavage site (shown in red). (B) Functional characterisation of DBS-S in S2 cells before and after UV light exposure. DBS-S, cleaved caspase-3 and DAPI are shown in the green/grey, red and blue channels, respectively. (C,D) Functional characterisation of DBS-S in *Drosophila* wing discs, expressing the pro-apoptotic protein Hid (C) or Hemipterous (D) activated under the regulation of *spalt*-Gal4 (SalG4) (DBS-S in green and cleaved caspase-3 in red). *spalt*-Gal4 is expressed in the central region of the wing pouch where the nuclei are positively labelled with the sensor. Shown in the panels are *xy* images of apical and basal focal planes of the wing discs and cross-sections of the epithelia (*z*-sections). The dashed yellow line indicates the location of the *z*-sections in the *xy* images. Blue and red arrowheads indicate apical and basal regions of the epithelium in the *z*-sections, respectively. White arrows point to cells apically located with activated DBS-S (i.e. nuclear GFP). (E) Physiological cell death triggered by cell competition mechanisms. Genetic Minute heterozygous mosaics (M/+ cells in blue) showing the colocalisation of nuclear DBS-S signal (green) and cleaved caspase-3 immunoreactivity (red) in presumptive apoptotic cells (red arrows). See Table S1 for a full description of the experimental genotypes displayed in all the figures.

Drice is fully activated by two sequential steps of enzymatic processing, with the first cleavage step being mediated by initiator caspases (mainly by Dronc; Fig S1A, Fig. 1A) (Lannan et al., 2007). Upon this first cleavage, Drice is split into two subunits (large and short), which remain strongly associated to form the active protease (Fig. S1A) (Lannan et al., 2007). We capitalised on this processing step to devise a reporter of initiator caspase activation, which will be hereafter referred to as the Drice-based sensor (DBS). As a foundation for the construct, we used an enzymatically inactive but still cleavable version of Drice: Drice^C211A^ (Fig. 1A) (Lannan et al., 2007). This construct configuration does not compromise the initiator caspase-mediated excision events but prevents undesirable activation of apoptosis (Lannan et al., 2007). We then appended the transmembrane domain of CD8 at the N terminus and a Histone2Av-GFP moiety to the C terminus (Fig. 1A). Following this design, we created two versions of DBS. One version included a full-length but mutated cDNA of Drice (CD8-Drice^C211A^-full length-Histone-GFP; hereafter DBS-FL), whereas the other contained a truncated template that only retained 16 amino acids downstream of the Dronc cleavage site (CD8-Drice^C211A^-short-Histone-GFP; hereafter DBS-S), which hypothetically prevents the interaction between the large and the small subunit of Drice (Fig. 1A). Both constructs were subcloned downstream of the ubiquitous *tubulin* promoter to assess their activity in S2 *Drosophila* cells and transgenic flies. In the absence of caspase activation, as expected, both constructs were anchored to the cellular membranes outside the nucleus (notice the predominant membrane GFP signal in 91% of the transfected cells; *n*=63 cells; Fig. 1B, Fig. S1B,C). However, 90 min after inducing cell death with UV light, the excision of the small Drice subunit in the DBS-S construct allowed the translocation of the Histone-GFP moiety into the nucleus within most of the transfected cells (78%; *n*=77; Fig. 1B, Fig. S1B). As confirmation that DBS-S faithfully reports on caspase activity, we found that the nuclear localisation of the Histone-GFP fragment was correlated with cleaved caspase-3 immunoreactivity (Fig. 1B). Additional evidence to rule out the possibility of non-specific cleavage of our sensor outside of the Drice template was obtained by observing that the fluorescent signal of DBS-FL remained attached to the membranes even in apoptotic conditions, highly likely because of the strong interaction between the large and small subunits of Drice (Fig. S1C). Equivalent observations were made when the sensor was expressed under the regulation of a different promoter (*actin* promoter; Fig. S1D). These results suggest that DBS-S is able to report on caspase activation in apoptotic cells, and there is no inadvertent or nonspecific cleavage of the DBS-S template without apoptotic stimuli.

### DBS-S is an early reporter of cell death in *Drosophila* cells

**Fig. 2.**
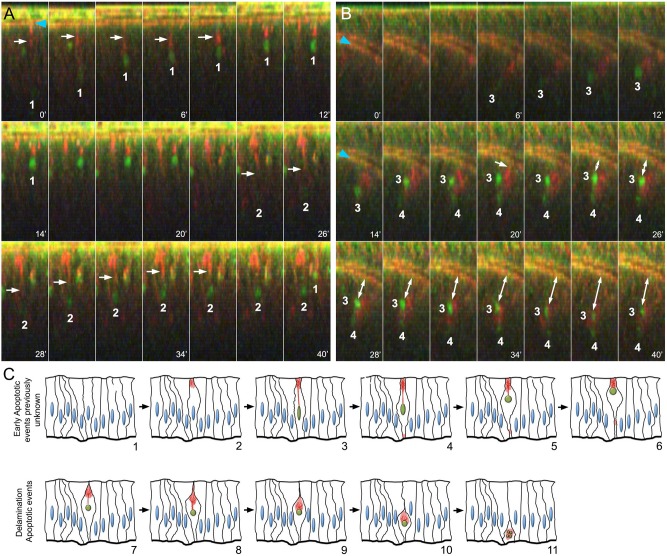
**Live imaging of imaginal discs upon irradiation.** (A) Time-lapse imaging of wing imaginal discs *ex vivo* (cross-sections) after irradiation (time is indicated in minutes; DBS-S is shown in green; lifeAct-Ruby shows actin in red). Notice the progressive accumulation of GFP signal in the nuclei of cells 1 and 2, the changes in their shape (roundness increase) and the movement towards the apical cell cortex. White arrows indicate the actin bundles connecting the apical cell cortex with the nuclei; notice the contraction of the actin bundle over time. The recording started 90 min after irradiation and lasted for 40 min. Snapshots were acquired every 2 min. (B) Delamination process of apoptotic cells 3 and 4 after completing apical migration; white arrow indicates apical cell detachment before delamination (20 min). Blue arrowheads indicate the normal accumulation of actin in the apical adherent cell junctions. Notice that actin remains accumulated around the nuclei during the movement towards the basal surface of the epithelium. Image acquisition conditions are described in A. Double-headed arrow indicates the basal delamination of DBS-S positive cells. (C) Schematic of early apoptotic events newly identified by DBS-S and lifeAct-Ruby in irradiated wing discs (1-6), as well as delamination events previously described during apoptosis (7-11). The activation of the reporter is indicated by the green nuclei, and the actin network is represented in red.

To investigate the activity of DBS-S in *Drosophila* tissues, we generated transgenic flies expressing the reporter under the regulation of ubiquitous promoters (*tubulin* or *actin*). The resulting transgenic flies were developmentally normal, fertile and morphologically indistinguishable from their wild-type siblings. These observations indicate that DBS-S expression did not compromise developmental processes that require either apoptotic cell death or non-apoptotic caspase activation (e.g. dorsal closure; Bischoff and Cseresnyes, 2009; Kanuka et al., 2005; Levayer et al., 2016; Marinari et al., 2012; Ninov and Martin-Blanco, 2007). Because wild-type imaginal discs have a low rate of spontaneous apoptosis in standard laboratory conditions, the nuclear labelling with DBS-S was extremely rare in this tissue (Fig. S1E). However, many nuclei were positively labelled in response to genetically induced apoptosis (Fig 1C,D, Fig. S2A), environmental apoptotic stimuli (Fig. 2, Fig. S2B,C), or physiologically triggered cell death (Fig. 1E, Fig. S2D) (Moreno et al., 2002). In pro-apoptotic conditions, cells positively marked with the DBS-S sensor usually had cleaved caspase-3 immunoreactivity (Fig. 1C-E) and were located at the basal surface of the epithelium (Fig. 1C,D). However, their signals did not necessarily colocalise at the subcellular level. This was expected to some extent, as cleaved caspase-3 immunoreactivity is mainly cytoplasmic, whereas the DBS-S signal accumulated in the nucleus. Interestingly, we also observed GFP-positive nuclei apically located in cells that had either low or no cleaved caspase-3 immunoreactivity (Fig. 1C,D, Fig. S1F). Similar results were obtained utilising all of the different transgenic lines expressing the construct (Fig. 1, Fig. S2). Western blot experiments also confirmed the cleavage of the sensor following the expected pattern upon apoptosis induction (Fig. S1G). These observations suggest that DBS-S is able to track the early stages of apoptosis, before compromised cells initiate the delamination process (Gudipaty and Rosenblatt, 2017), and confirm the specific cleavage of the sensor in response to apoptotic stimuli in either induced or physiological situations. Next, we investigated the performance of DBS-S in non-apoptotic scenarios, analysing its activation within the proneural clusters of the wing discs (Kanuka et al., 2005). However, no nuclear fluorescent signal was observed during the course of these experiments (not shown). These results suggest that DBS-S only detects the high levels of caspase activation that accompany apoptotic cells.

### Apical nuclear migration in pre-apoptotic cells uncovered by DBS-S

After completing the basic characterisation of DBS-S in fixed samples, we analysed the performance of the sensor in live tissues. DBS-S readily identified apoptotic cells in imaginal discs filmed *ex vivo* upon irradiation (Movie 1). Strikingly, we observed that most of the GFP-positive nuclei aligned to the apical surface soon after irradiation (Fig. 2A, Fig. S2C, Movies 1-3). This movement was tightly correlated with the apical accumulation of actin (Fig. 2A, Movies 2, 3). Indeed, the contraction of actin bundles appeared to pull the nuclei up towards the apical cell cortex, whilst the nuclei kept accumulating the GFP signal (Fig. 2A, Fig. S2C, Movies 2, 3). GFP-positive nuclei also changed their shape during the apical migration, progressively becoming more rounded (Fig. 2A, Fig. S2C, Movies 2, 3). Subsequently, during the delamination process, the nuclei were pushed towards the basal side of the epithelium (Fig. 2B, Movie 4) along with the actin-enriched structure (Fig. 2B,C, Movie 4). During most of the delamination process, the nuclei remained intact (Fig. S1E); therefore, nuclear fragmentation appears to be a characteristic event of apoptotic cells in the final stages of delamination. A similar association between nuclei and polymerised actin was also noticed in apoptotic S2 cells (Fig. S1D). These results confirmed that DBS-S is an early sensor of apoptosis, which in turn has allowed us to uncover unknown behaviours of pre-apoptotic nuclei (Fig. 2C, Movie 5). DBS-S was also an effective marker of apoptotic cells in other tissues beyond the wing discs. DBS-S marked characteristic apoptotic larval epidermal cells during pupal stages (Fig. S2D, Movie 6) (Ninov et al., 2007) and apoptotic histoblasts (Movie 7) of which 3% die during metamorphosis (Bischoff and Cseresnyes, 2009). Exceptionally in embryos, either physiological or experimental induction of apoptosis (with Ci-gal4 and UAS-reaper) did not lead to activation of the DBS-S sensor (not shown). However, this uncertain disparity does not undermine the power of the DBS-S to identify apoptotic cells in most situations.

### Sensitivity of DBS-S to initiator caspases and P35

**Fig. 3.**
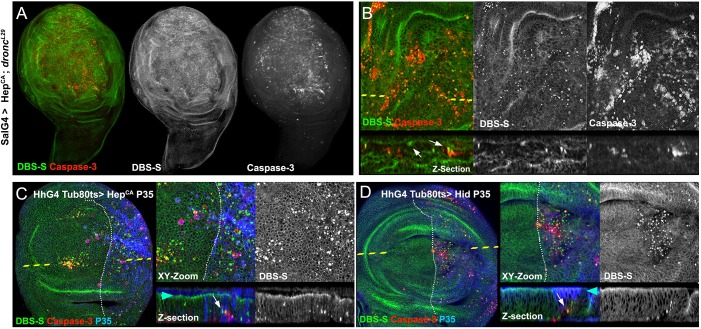
**Behaviour of DBS-S in compromised cells that cannot complete the apoptosis programme.** (A) Wing cells mutant for *Dronc* that ectopically express an activated form of Hemipterous under the control of *spalt*-Gal4 (SalG4) (DBS-S in green, cleaved caspase-3 in red). (B) Magnifications of the images shown in A. Notice the presence of cells positively labelled with DBS-S (nuclear signal of the reporter in green; white arrows) and cleaved caspase-3 immunoreactivity (red). (C,D) Identification and labelling of undead cells in wing imaginal discs expressing simultaneously Hemipterous activated and P35 under the control of Hedgehog-Gal4 (C) or simultaneously expressing Hid and P35 under the control of Hedgehog-Gal4 (D). *xy* images, high magnifications and cross-sections of wing epithelium (*z*-sections) are shown. DBS-S is shown in green, cleaved caspase-3 staining in red and P35 immunostaining in blue. The anterior (left side) and posterior (right side) compartments are separated by the discontinuous white line. The discontinuous yellow line indicates the location of the *z*-section in the *xy* image. White arrows indicate delaminated cells positively labelled with DBS-S (green). Blue and red arrowheads indicate the apical and basal parts of the wing epithelium, respectively.

Although *Dronc* is considered the main initiator caspase in *Drosophila*, other initiator caspases (*Dredd* and *Strica*) can also contribute to Drice activation. To address whether Dronc is the sole activator of DBS-S, we genetically induced apoptosis in a *Dronc* null mutant background (*Dronc*^L29^) by ectopically activating JNK signalling. Imaginal discs of such genetic conditions are phenotypically hyperplastic and still show a limited number of cleaved caspase-3-positive cells (Perez-Garijo et al., 2009). Confirming previous results, the immunoreactivity of cleaved caspase-3 and the nuclear GFP from DBS-S were observed in the same cells (Fig. 3A,B), although both signals were located in different subcellular compartments. Importantly, these findings confirm that other initiator caspases in addition to *Dronc* could mediate the cleavage of Drice, and therefore the DBS-S template, in pro-apoptotic conditions (Xu et al., 2005).

Next, we investigated the impact of the effector caspase inhibitor P35 on DBS-S. P35 covalently binds to the catalytic residue of effector caspases compromising their ability to cleave substrates (Hay et al., 1994; LaCount et al., 2000; Lannan et al., 2007). Accordingly, it also prevents the activation of effector caspase reporters (Bardet et al., 2008; Ding et al., 2016; Florentin and Arama, 2012; Schott et al., 2017; Takemoto et al., 2003; Tang et al., 2015; To et al., 2015). In these experiments, we used both Hemipterous (an activator of JNK signalling) and Hid as inducers of apoptosis. As P35 acts downstream of *Dronc*, it did not prevent the nuclear import of GFP (Fig. 3C,D). As expected, this indicates that DBS-S is insensitive to inhibitors of the effector caspases. Additionally, it supports the notion that DBS-S cleavage is primarily mediated by initiator caspases (see next section). This key feature brings up the possibility of identifying caspase-dependent processes with our sensor that do not require the entire caspase cascade (e.g. tracking of the so-called ‘undead cells’; Huh et al., 2004; Perez-Garijo et al., 2004; Ryoo et al., 2004; Fig. 3C,D).

### DBS-S-QF: a temporal caspase reporter that allows the genetic manipulation of caspase-activating cells

**Fig. 4.**
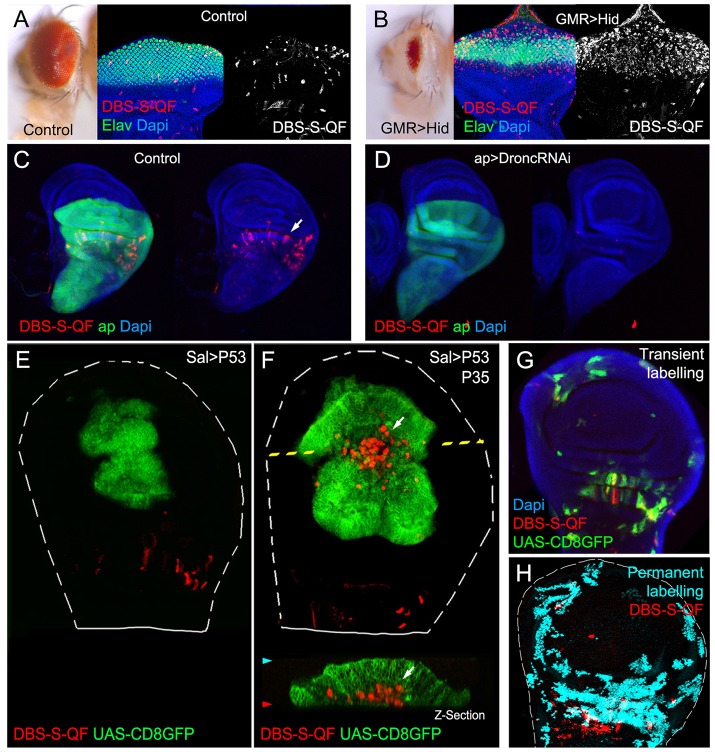
**Functional characterisation of the DBS-S-QF sensor.** (A,B) Activation of the DBS-S-QF reporter in either wild-type (A) or apoptotic eyes ectopically expressing Hid under the regulation of GMR promoter (B). Adult eyes and imaginal discs are shown on the left and right panels, respectively. Activation of DBS-S-QF is shown in red upon induction of a QUAS-Tomato-HA transgene (anti-HA in red). Differentiated regions of the eye express the neuronal identity marker Elav (green); nuclei are labelled with DAPI. (C) Activation of DBS-S-QF (anti-HA in red; white arrow) in a wild-type wing disc expressing CD8-GFP (UAS-CD8-GFP) under the regulation of *apterous*-Gal4 (green); the experiment was carried out at 29°C. (D) Activation of DBS-S-QF (anti-HA in red) in a wing disc expressing a RNA interference against *Dronc* (UAS-Dronc-RNAi) and CD8-GFP under the regulation of *apterous*-Gal4 (green); the experiment was performed at 29°C. (E) Overexpression of P53 and CD8-GFP under the regulation of *spalt*-Gal4 (green); red channel shows the DBS-S-QF activation (QUAS-Tomato-HA, anti-HA in red). Notice the absence of Tomato expression in the GFP-expressing cells. The dashed white line outlines the wing disc. (F) Co-expression of P53, P35 and CD8-GFP in the *spalt*-Gal4 expression domain (green); red channel shows the DBS-S-QF activation (QUAS-Tomato-HA, anti-HA in red); white arrow in *xy*-image or *z*-section indicates the appearance of ‘undead cells’ basally delaminated (‘undead cells’ refers to caspase-activating cells that cannot finalise the apoptosis programme owing to the presence of P35 blocking the activation of the effector caspases). (G) Temporal perspective of caspase-activating cells in a wild-type wing disc using transient fluorescent markers expressed under the control of DBS-S-QF; QUAS-Tomato-HA (anti-HA in red) labels ongoing caspase activation; old caspase activation is shown in green (QUAS-Gal4 UAS-mCD8-GFP); recent-past caspase activation is indicated by the colocalisation of both markers (yellow); DAPI labels nuclei. (H) Lineage tracing of caspase-activating cells in a wild-type wing disc obtained with DBS-S-QF (ongoing activation of DBS-S-QF is shown in red; nuclear β-gal staining shows in cyan the permanent labelling of caspase-activating cells).

**Fig. 5.**
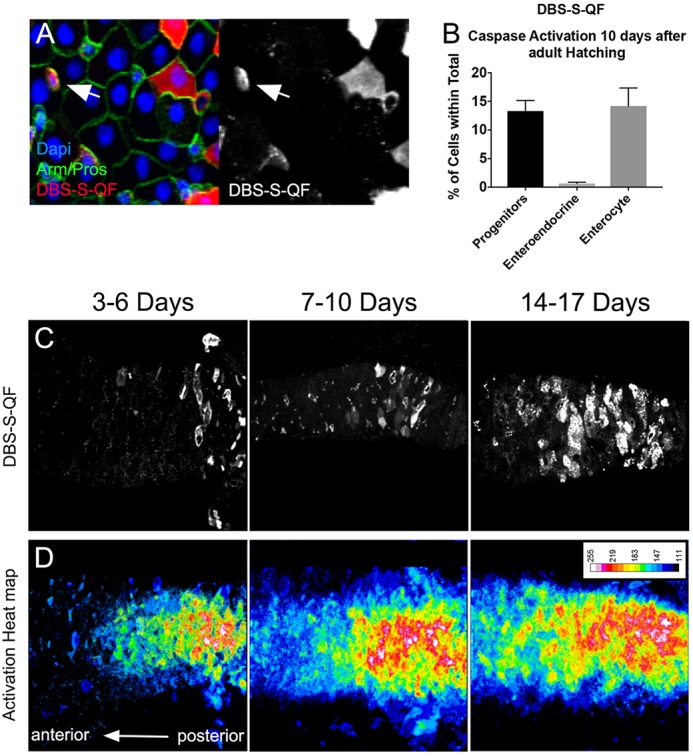
**Functional characterisation of DBS-S-QF in the posterior midgut of mated female adult flies.** (A) Transient expression of DBS-S-QF in the midgut (QUAS-Tomato-HA; anti-HA in red); anti-Armadillo staining in green labels the cell membranes; nuclear anti-Prospero staining in green labels enteroendocrine cells; DAPI staining marks nuclei. White arrows point to intestinal progenitor cells (intestinal stem cells or enteroblasts) positively labelled by DBS-QF. (B) Quantification of cell types transiently activating DBS-S-QF in the posterior midgut. Large nuclear cell size was the criteria to assign enterocyte cell identity. The criteria applied to identify gut progenitor cells (either intestinal stem cells or enteroblasts) was the small cell size and negative immunolabelling against Prospero. *n*=10. Error bars indicate s.e.m. (C,D) DBS-S-QF activation in the gut at different time points during adulthood. C shows DBS-S-QF activation, with QUAS-Tomato-HA, anti-HA in white. D shows the overlay of multiple guts at the same time point. Warmer areas (red) in the heat map indicate higher concentration of cells activating the sensor, whereas colder areas (blue) areas reflect less cells activating the reporter. DBS-S-QF: 3-6 days *n*=13; 7-10 days *n*=25; 14-17 days *n*=24.

To increase the sensitivity and broaden the usefulness of our sensor, we decided to replace the Histone-GFP-encoding fragment in DBS-S with the transcriptional activator QF (Potter et al., 2010) (DBS-S-QF; Fig. S3A). As previously shown in DBS-S transgenic flies, the constitutive expression of DBS-S-QF did not generate morphological or developmental defects, thus ruling out either toxic or inadvertent side effects of the construct. Likewise, the DBS-S-QF reporter retained most of the features of DBS-S, such as the labelling of apoptotic cells (Fig. 4A,B, Fig. S3B,C). However, the labelling of apoptotic cells with DBS-S-QF is strongly linked to the properties of the cellular marker that is induced (e.g. P53-expressing cells in the wing can be easily labelled with the QUAS-*lacZ* transgene, but it is harder to label with QUAS-Tomato-HA; compare Fig.S3B with S3D). This feature was also highly tissue specific (e.g. P53-expressing cells activated the QUAS-Tomato-HA transgene in the eye imaginal discs but failed to do so in the wing discs; compare Fig. S3C with S3D; see Discussion). Importantly, the basal activation of DBS-S-QF also disappeared when expression of *Dronc* was eliminated (Fig. 4C,D)*.* Conversely, P35 expression did not prevent the cleavage of DBS-S-QF, allowing ‘undead’ cells to be tracked (Fig. 4F; notice in this instance the activation of QUAS-Tomato in comparison with controls in Fig. 4E; see Discussion). By virtue of releasing the transcription activator QF, DBS-S-QF was also able to detect caspase activation in non-apoptotic contexts (sensory organ precursors; Fig. S3E). These results collectively confirm the initiator caspase-dependent cleavage of the DBS template in apoptotic and physiological conditions. The QF release upon caspase cleavage from DBS-S-QF enables the genetic manipulation of caspase-activating cells in combination with QUAS-Gal4/UAS transgenes (Fig. 4C-F, Fig. S3E-G), as well as their long-lasting labelling (Fig. S3H-J). Taking advantage of this feature, we replicated previously described patterns of caspase activation in wing imaginal discs (Ding et al., 2016; Tang et al., 2015) (Fig 4G,H, Fig. S3J) and we also observed striking differences in other tissues such as the posterior midgut of mated female adult flies. Whereas effector caspase-based sensors were exclusively activated in enterocytes throughout the posterior midgut (Ding et al., 2016; Tang et al., 2015), DBS-S-QF was also activated within intestinal progenitor cells (intestinal stem cells and enteroblasts; Fig. 5A,B, Fig. S4A). Additionally, although this activation was initially localised in the posterior region of the midgut (R4c region; Buchon et al., 2013), the activation spread anteriorly over time (R4a and R4b; Fig. 5C,D, Fig. S4A). Importantly, sensor-positive cells did not show signs of apoptosis and remained in the epithelium (Fig. S4B). Beyond confirming the presence of widespread non-lethal caspase activation (Ding et al., 2016; Tang et al., 2015), our results suggest the existence of stereotyped patterns of initiator caspase activation, which are likely to be regulated by either developmental or environmental cues in the *Drosophila* gut.

## DISCUSSION

The labelling of caspase-activating cells in live tissues is crucial for identification of caspase-dependent biological processes in apoptotic and non-apoptotic cellular scenarios. We have developed a new set of *Drosophila* caspase sensors that provide a complementary view of caspase activation in comparison with currently available reporters (Xu et al., 2018), through direct monitoring of the activation of initiator caspases. Our sensors are initially retained at the cellular membranes through a CD8 transmembrane domain and exclusively, upon initiator caspase activation (mainly Dronc), allow the release of either a Histone-GFP fragment or the transcriptional activator QF. Although our sensors effectively report on caspase activation in most of the tissues analysed, they are not universal (e.g. DBS-S does not work in embryos). The origin of this disparity is uncertain but it could be linked to the fact that they are attached to the intracellular membranes via a CD8 fragment. Because different upstream protein complexes can activate initiator caspases in specific subcellular locations (Amcheslavsky et al., 2018; Kang et al., 2017), the membrane retention domain could limit the interaction between our sensors and active initiator caspases in particular cell types. Beyond the stated limitations, the design of our caspase reporters has revealed unknown properties of caspase-activating cells in cellular scenarios that do not demand the activation of the entire caspase cascade.

### Novel apoptotic steps uncovered by DBS-S: apical nuclear migration of pre-apoptotic cells

Since the early 1970s, nuclear shape changes have been considered hallmarks of apoptosis, distinguishing this process from other programmes of cell death (such as necrosis or autophagy) (Kerr et al., 1972; Prokhorova et al., 2015). Indeed, nuclear shrinking and fragmentation are indicative of chromatin condensation and DNA degradation during apoptosis (Prokhorova et al., 2015). In *Drosophila*, these events occur relatively late and are tightly coordinated with the delamination process (Gudipaty and Rosenblatt, 2017; Kawamoto et al., 2016; Ohsawa et al., 2018) (Fig. 2C, delamination phases from step 7 onwards). Unsurprisingly, cell delamination and nuclear fragmentation are decisively influenced by caspase-mediated actin cytoskeleton rearrangements (Gudipaty and Rosenblatt, 2017; Kawamoto et al., 2016; Prokhorova et al., 2015). Our work now uncovers another actin-dependent event that occurs earlier during apoptosis. As the nuclear GFP signal from DBS-S accumulates, the nuclei are seen to undergo an apical migration and shape changes (an increase in roundness) (Fig. 2A,C, Movie 5). It is also tightly linked to the apical accumulation of actin and the subsequent contraction of an actin bundle connecting the apical cell cortex with the nucleus (Fig. 2A,C, Movie 5). These new apoptotic events precede the distinctive basal delamination movement of dying cells and, therefore, can be considered the earliest subcellular manifestation of apoptosis described to date in *Drosophila* cells. Interestingly, the uncovered apical migration of pre-apoptotic nuclei bears remarkable similarities to the interkinetic nuclear movement that normally occurs before mitosis (Meyer et al., 2011). However, the actin enrichment as well as the actin-mediated events appear to be localised in opposing subcellular domains in the two processes. Whereas the actin-dependent basal cellular contraction pushes nuclei towards the apical cortex during mitosis (Meyer et al., 2011), the retraction of apical actin bundles appears to pull nuclei upwards during apoptosis. Incidentally, the caspase-dependent cytoskeleton rearrangements that presumably facilitate the interkinetic nuclear migration could also be linked with the mechanical forces responsible for the epithelial folding in specific tissues (Monier et al., 2015). Although it is still premature to establish the biological significance of this pre-apoptotic apical migration, we hypothesise that it could facilitate the rapid chromatin condensation and shutdown of gene expression, similar to the process occurring during mitosis (Zaret, 2014). Collectively, these events could inhibit essential cellular functions earlier than previously thought and before the DNA degradation is completed, thus ensuring the efficient elimination of apoptotic cells.

### Suitability of our sensors to detect caspase-related processes independent of effector caspases: implication of caspases during cell delamination

In contrast to published sensors (Bardet et al., 2008; Ding et al., 2016; Florentin and Arama, 2012; Schott et al., 2017; Takemoto et al., 2003; Tang et al., 2015; To et al., 2015), our reporters are insensitive to inhibitors of the effector caspases. This feature has enabled for the first time the specific detection of initiator caspase activation in cells in which the completion of the apoptotic programme was experimentally prevented (e.g. ‘undead cells’). Indeed, visualisation of ‘undead cells’ with DBS-S-QF has allowed us to demonstrate that, even though these cells do not undergo apoptosis, they can still delaminate (Fig. 3C,D, Fig. 4F). This suggests that the threshold of caspase activation required for cell delamination is lower than that for organelle destruction during apoptosis. It also suggests that cell delamination could be exclusively driven by initiator caspases. This observation is, however, very puzzling as it is known that effector caspases are essential for physiological cell delamination in the thorax (Levayer et al., 2016; Marinari et al., 2012). One possibility is that initiator caspase activation is much stronger in our experimental conditions owing to the overexpression of pro-apoptotic factors and is consequently sufficient for cell delamination. Alternatively, the process of caspase-mediated cellular delamination could be different depending on the tissue architecture.

### The faster you proliferate, the quicker you disappear

DBS-S takes advantage of rapid changes in the subcellular localisation of the GFP signal to label caspase-activating cells, whereas the QF version of the sensor demands a more complex sequence of events (e.g. QF translocation to the nucleus, transcriptional activation of the reporter gene and protein maturation). These additional steps confer higher sensitivity to DBS-S-QF, but inherently delay the labelling of apoptotic cells. Indeed, P53-apoptotic cells in the wing can be always detected with DBS-S but only using specific cellular markers with DBS-S-QF, presumably because dying cells are eliminated before they have opportunity to express the readout gene of choice (whereas P53-expressing cells of the wing can activate QUAS-*lacZ*, they failed to induce the expression of QUAS-Tomato-HA; compare Fig. S3B with S3D). Supporting this hypothesis, the co-expression of P35 with P53 prevented or slowed down the elimination of caspase-activating cells in the wing and consequently allowed the induction of the QUAS-Tomato-HA signal under the regulation of DBS-S-QF (compare Fig. 4E and 4F). Interestingly, similar differences were observed between P53 fast-proliferating cells of the wing and non-proliferative cells behind the morphogenetic furrow of the eye (compare Fig. S3C and S3D). Therefore, we conclude that the ability of DBS-S-QF to label apoptotic cells largely depends on the disposal rate of apoptotic cells and the marker of choice induced by DBS-S-QF activation. Furthermore, they illustrate the coordination between cell proliferation and cell destruction events previously hypothesised (Abrams and White, 2004).

### Initiator versus effector caspase activation patterns

The spatiotemporal pattern of caspase activation revealed by our reporters and other ‘historical sensors’ (Ding et al., 2016; Tang et al., 2015) show remarkable similarities and differences (Fig. 5). The similarities are not surprising as the activation of previously described sensors demand the enzymatic activity of initiator caspases. In this sense, our sensors would incorporate the activation patterns observed with effector caspase reporters. In this regard, the preferential clonal expansion of caspase-activating cells in the proximal regions of the wing hinge compared with the distal parts is interesting (Fig. S3J). This could be associated with the enrichment of these territories in sensory organ precursor cells activating our sensor. Importantly, caspase activation in these areas has been shown to modulate their growth (Ding et al., 2016).

The discrepancies in the gut and potentially other tissues could be a consequence of initiator caspase-mediated cleavage of our Drice template in processes that do not require the activation of effector caspases. Importantly, these disparities could be exploited to obtain specific information regarding such biological processes.

### Anastasis and non-apoptotic caspase functions

Our results using DBS-S-QF confirm the widespread and non-apoptotic caspase activation previously observed in many *Drosophila* organs (Ding et al., 2016; Tang et al., 2015). However, several factors suggest this is likely to be related to the regulation of specific cellular functions, instead of the anastasis phenomenon (in which cells activate caspases to trigger the apoptosis programme but eventually revert the course of events without completing cell death; Ding et al., 2016; Tang et al., 2015). Supporting this hypothesis, we find non-lethal caspase activation in a large number of cells in different tissues without signs of apoptosis (Fig. 4F, Fig. 5). Furthermore, the patterns of non-apoptotic caspase activation observed with DBS-S-QF can be highly stereotyped, thus suggesting developmental control, as opposed to a response to random insults (Fig. 4H, Fig. 5, Fig. S4). Nevertheless, further functional studies will be needed in order to establish the biological functions coupled to such non-apoptotic patterns of caspase activation in different organs.

In summary, we have described the functional characterisation of novel initiator caspase sensors that open up experimental opportunities to uncover new biological features of caspase-activating cells in either apoptotic or non-apoptotic scenarios.

## MATERIALS AND METHODS

### Fly strains and experimental genotypes

All fly strains used are described at www.flybase.org unless otherwise indicated. All of our experiments were carried out at 25°C unless otherwise indicated. A full description of experimental genotypes can be found in Table S1.

### Molecular cloning and generation of new fly strains

All PCRs were performed with Q5 High-Fidelity polymerase from New England Biolabs (NEB, M0492L). Standard subcloning protocols and gene synthesis (Genewiz) were used to generate all the DNA constructs. New transgenic lines expressing our constructs were obtained by random P-element transgenesis and attP/attB PhiC31-mediated integration. Bestgene injected the DNA plasmids in *Drosophila* embryos in order to generate the new transgenic strains. Most of the plasmids designed for this study will be deposited at the *Drosophila* Genomics Resource Center (DGRC), IN, USA. Similarly, new fly strains generated will be deposited at the Bloomington *Drosophila* Stock Center, IN, USA. Until resources transfer is completed, sequences and reagents will be provided upon request.

#### Drice-based sensor-histone-GFP constructs

A Bluescript vector containing DNA encoding the N-terminal region (transmembrane domains) of mouse CD8 protein was used as a starting point. DNA encoding both the Drice mutant template (Drice C211A cDNA was a gift from Dr Paul D. Friesen, University of Wisconsin-Madison, USA) and the Histone-2AV GFP were amplified by PCR and cloned into ToPo-TA vectors. The Histone-2AV-GFP was then inserted at the unique *Bgl*II-*Xba*I sites of the Bluescript vector lying after the CD8. The full-length and short Drice mutant templates were then inserted into the unique *Age*I-*Bgl*II sites present between the CD8 and the Histone-2AV-GFP. Each construct was finally cloned as a *Not*I-*Xba*I fragment downstream of either *tubulin* or *actin* promoters included in a customised pCasper vector (this vector contains an attB-recombination site, a multicloning site and the fly selection marker white +). Random P-element insertions generated the DBS-S strains located in the II and III chromosomes, respectively. The integration of the construct on the X chromosome was obtained by using PhiC31-mediated recombination. The selected attP-landing site for generating the transgenic line was 9753.

#### DBS-S-QF reporter construct

To generate the QF version of DBS-S, the histone-2AV-GFP fragment was replaced by the open reading frame (ORF) of the transcriptional activator QF. We introduced a HA-motif at the N terminus of the QF ORF and a *Bam*HI restriction site via PCR. An extra *Spe*I site was also appended to the C terminus of the PCR product. Addgene plasmid #46134 (deposited by Christopher Potter) was used as template for obtaining the PCR fragment. The PCR product was then subcloned in the unique *Bgl*II-*Xba*I sites present in DBS-S-QF. The whole construct DBS-S-QF was finally transferred into an *actin* attb vector as a *Not*I-*Sal*I fragment. The construct was integrated on the X chromosome using PhiC31-mediated recombination. The selected attP-landing site for generating the transgenic line was 9753.

#### Actin mitoPlum-2A-lifeAct-Ruby-2A-DBS-S construct

We first generated a pMTV5 vector containing two repeated copies of the 2A peptide spaced conveniently with restriction sites (pMTV5 configuration *Not*I-*Bgl*II-2A-*Avr*II-*Cla*I-2A-*Xma*I-*Xba*I). Before the first 2A peptide we subcloned a plum fluorescent protein tagged with a mitochondrial localisation signal. Gene synthesis and subsequent cloning as a *Not*I-*Bgl*II fragment was the strategy followed for the cloning of the Plum product. In between the two 2A peptides, we cloned the PCR fragment encoding the lifeAct-Ruby protein (the template was a gift from Dr E. Ober) using the unique sites *Avr*II-*Cla*I sites. After the second 2A peptide, we cloned DBS-S as a fragment *Xma*I-*Xba*I. The whole construct Actin mitoPlum-2A-lifeAct-Ruby-2A-DBS-S was finally subcloned into an *actin* attb vector as a *Not*I-*Xba*I fragment. The construct was integrated on the III chromosome using PhiC31-mediated recombination. The selected attP-landing site for generating the transgenic line was 9732.

#### QUAS-Gal4 construct

First, we extracted the Gal4 ORF from pMTV5 Gal4 as a *NotI*-*Avr*II fragment. Subsequently, the fragment was subcloned into a QUAS-attB vector containing a customised multicloning site. The construct was integrated on the III chromosome using PhiC31-mediated recombination. The selected attP-landing site for generating the transgenic line was 9748.

#### Primer description and sequences

Forward Histone2AV-GFP with *Bgl*II site: GATC**AGATCT**GCTGGCGGTAAAGCAGGCAA; Reverse Histone2AV-GFP with *Xba*I site: GATC**TCTAGA**TTATTTGTATAGTTCATCCAT; Forward Drice with *Age*I site: GATC**ACCGGT**GACGCCACTAACAATGGAGAATCCGCCG; Reverse Drice full length with *Bgl*II site: GATC**AGATCT**AACCCGTCCGGCTGGTGCCAACTGCTTGTCGC; Reverse Drice short with *Bgl*II site: GATC**AGATCT**GTGCACTGGAATCTTGTAGCTCATCG; Forward HA-QF with *Bam*HI site: TTACAC**GGATCC**AAGCTTTACCCATACGACGTCCCTGACTATGCGCCTCCGGGAATTGGGAATTCCAACATGCCGCCTAAACGCAAGACACTCAATGC; Reverse QF polyA with *Spe*I site: TTTATATA**ACTAGT**GGATCTCTAGAGGTACCCTCGAGCCGCGGCCGCGGATCTAAACGAGTTTTTAAGCAAACTCACTCCCTGACAATAAAAACGC; Forward lifeAct-Ruby with *Avr*II site: TCCGGC**CCTAGG**AATGGGTGTCGCAGATTTGATCAAGAAATTCGAAAGCATCTCAAAGGAAGAA; Reverse lifeAct-Ruby with *Cla*I site: TGCCCA**ATCGAT**AGAGCGCCTGTGCTATGTCTGCCCTCAGC; Forward CD8 with *Xma*I site ATGCGT**CCCGGG**ATGGCCTCACCGTTGACCCGCTTTCTGTCGC; Reverse Histone-2Av-GFP with *Xba*I site: TAAGGG**TCTAGA**TTATTTGTATAGTTCATCCATGCC. Enzyme restriction sites are in bold.

### Mosaic analysis

Female *y w hsp70-flp*; FRT40A *M arm*-lacZ; DBS-S were crossed with male FRT40A to obtain +/+ genetic mosaics in a M/+ genetic background, thus triggering cell competition. Progeny of this cross were heat-shocked at 48-72 h after egg laying at 37°C for 1 h. The larvae were dissected before puparation to perform the immunostainings in Fig. 1E.

### Temperature-shift experiments

After 12 h of egg laying at 25°C, crossed flies were transferred into new tubes. Eggs laid were kept at 22°C for 3 days in order to prevent lethality at early developmental stages. Hatched larvae were then transferred to 25°C until dissection before puparium formation. The genotypes of the flies used in these experiments were: w; *UAS-Hep.Act, Tubulin-DBS-S/TubG80^ts^; Hedgehog-Gal4/UAS-P35* (Fig. 3C); w; *UAS-Hid, Tubulin-DBS-S /TubG80^ts^; Hedgehog-Gal4/UAS-P35* (Fig. 3D).

### Immunohistochemistry

Immunostainings and washes of imaginal discs and S2 cells were performed according to standard protocols [fixing in PBS 4% paraformaldehyde, washing in PBT 0.3% (0.3% Triton X-100 in PBS)]. Adult *Drosophila* intestines were dissected in ice-cold PBS. To fix, wash solution (0.7% NaCl, 0.05% Triton X-100) was heated to approximately 90°C and the intestines submerged into this solution for 6 s. Following this, the intestines were rapidly cooled through submersion in ice-cold wash solution. Three rapid washes in PBT (0.3%) preceded blocking in 1% bovine serum albumin in PBT (0.3%) for at least 1 h. For imaginal disc immunostaining, primary and secondary antibody incubations were performed at room temperature for 3 h. For gut immunostaining, overnight incubation at 4°C with the primary antibodies and 2 h at room temperature with the secondary antibodies was necessary. Primary antibodies used in our experiments were: anti-cleaved Caspase-3 (1:100; Cell Signaling, 9661); anti-P35 (1:500; Imgenex, IMG5740); rabbit anti-HA (1:1000; Cell Signaling, C29F4); mouse anti-β-Gal (1:500; Promega, Z378B); chicken anti-β-Gal (1:200; Abcam, AB9361); anti-Armadillo (1:50; Developmental Studies Hybridoma Bank, N2 7A1, armadillo-c); anti-Elav (1:200; Developmental Studies Hybridoma Bank; 9F8A9); anti-Prospero (1:20; Developmental Studies Hybridoma Bank, MR1A). Conjugated secondary antibodies were diluted in 0.3% PBT and used in a final concentration (1:200): anti-mouse Alexa 555, anti-mouse Alexa 488, anti-mouse Alexa 647, anti-rabbit Alexa 555, anti-rabbit Alexa 633 (Molecular Probes, Invitrogen; A-31570, A-21202, A-31571, A-31572, A-21072, respectively). Following incubation in secondary antibodies, samples were washed in PBT several times for 60 min and mounted in Vectashield with DAPI (1:1000; Thermo Scientific, 62248).

### Irradiation protocol

Larvae were exposed to a source of γ-ray during the irradiation experiments. Time exposure was adjusted for administrating a dose of 1500 rads to the samples.

### Western blot

Protein extracts were obtained from either non-irradiated or irradiated larvae (4 h post-treatment). We then followed standard protocols for processing samples and immunoblotting the membranes. We used anti-GFP (1:3000; Sigma, 11814460001) as primary antibody in these experiments.

### Cell culture and transfection of S2 and S2-R+ cells

S2 and S2-R+ cells were obtained from the DGRC public repository. Cells were grown in Gibco Schneider's medium supplemented with 1% L-glutamine, 10% fetal calf serum and antibiotics (1% penicillin/streptavidin). In all of the experiments, 1 µg of plasmid was transfected using Effectene (Qiagen) reagent, following manufacturer's instructions. Induced apoptosis was achieved after exposing the cells for 10 min to UV light (dose 200 mJ/cm^2^ UVC) using plates and cover slips transparent to UV light. Upon UV light treatment, cells were then fixed and immunolabelled at different time points (1.5 h and 4 h). In most experiments, cells were grown on normal coverslips placed in 6-well plates; however, coverslips coated with poly-lysine (Sigma, P0425-72EA) were used to facilitate the visualisation of cell projections and active actin in Fig. S1E.

### Imaging of fixed and live samples

Fluorescent imaging of fixed and live imaginal discs was performed with a Leica SP8 laser-scanning confocal microscope using LAS AF software. *Drosophila* intestinal fixed samples were imaged using the Olympus Fluoview FV1200 and associated software. *z*-stacks were taken with a 40× objective at intervals along the apical-basal axis that ensured adequate resolution in the *z* dimension (step size 0.5-1.5-μm). Acquired images were then processed using ImageJ and Adobe Photoshop CS6. Culture conditions and live recording of imaginal discs was performed as described by Mao and collaborators (Mao et al., 2011). Ninety minutes after irradiation, wing imaginal discs were filmed for 40-60 min. Four to six focal planes covering the subapical region of the wing disc epithelia were projected using ImageJ in order to obtain full representation of the nucleus and actin projections from single cells (Movie 1). The ‘Reslice’ function of Image J. was used to generate cross-sectional movies (Movies 2-4); again, four to six focal planes were merged in order to obtain full representation of the nucleus and actin projections from single cells.

4D microscopy of the abdominal epidermis was performed with a Leica SP8 confocal microscope at 25±1°C. Pupae were staged according to Bainbridge and Bownes (1981) and then were dissected and filmed as described by Seijo-Barandiaran et al. (2015). A step size of 2 or 2.5 µm in *z* was used during the filming to image the samples. Samples were imaged using a time interval range of 120 s to 180 s, depending on the experiment.

### Plot generation and ImageJ workflows

To estimate the intensity of the nuclear GFP signal from DBS-S sensor and the cleaved caspase-3 immunostaining along the apico-basal axis (Fig. S1F), we applied the following workflow in Fiji (ImageJ) software. We captured images along the apico-basal axis of different imaginal discs at the same magnification and laser intensity. Then the signals from the different colour stainings were combined and transversal sections were generated using the reslice plugin from ImageJ software. Transversal sections were then converted to 8 bit and subsequently thresholded to generate black and white images. The tool line with a specific thickness of 450 was trace from the apical part of the section to the basal side in the same position for each channel (DBS-S signal or cleaved caspase-3), before applying the plot profile function from the menu.

Heatmaps in Fig. 5 were produced using Fiji (ImageJ) software. Original images were acquired with the same orientation and magnification. The enriched area in stem cells of the R5 segment was used as reference to align the guts. On average, 20 focal planes per gut, containing all the information from the apical to the middle sections of a *z*-stack, were projected using the ‘Average intensity’ function. Subsequently, all the channels not containing information related to reporter activation were discarded. The projected reporter channel images were combined into a new single *z*-stack of projections, before applying an average intensity projection again. Following this. we applied the 16-colour LUT. An equivalent workflow was applied to obtain the accumulative plot of clones formed by cells permanently labelled with DBS-QF (Fig. S3J).

The dot plots in Fig. S4 were produced using Fiji (ImageJ) software. Dot plots were created by combining all of the channels (RGB) into one channel. Following this, a new black background channel was inserted. A composite image was selected and the pencil tool used to mark (in white) the location of presumptive progenitor cells onto the black channel. The nuclear size and Armadillo/Prospero immunostaining were used to assign the cell fate to different intestinal cells. A projection of this channel was created and for quantification purposes the dots were separated on the projected image. Three equal sections of the resultant picture were counted sequentially using the ‘Analyse particle’ plugin of ImageJ and the percentage of enteroblasts in each location was calculated.

## Supplementary Material

Supplementary information
