# Supplementary material for: Novel initiator caspase reporters uncover previously unknown features of caspase-activating cells
Source: Development. 2018 Dec 4;145(23):dev170811. doi: 10.1242/dev.170811 (PMC6288387; doi:10.1242/dev.170811)
Supplement: Supplementary information [file develop-145-170811-s1.pdf]

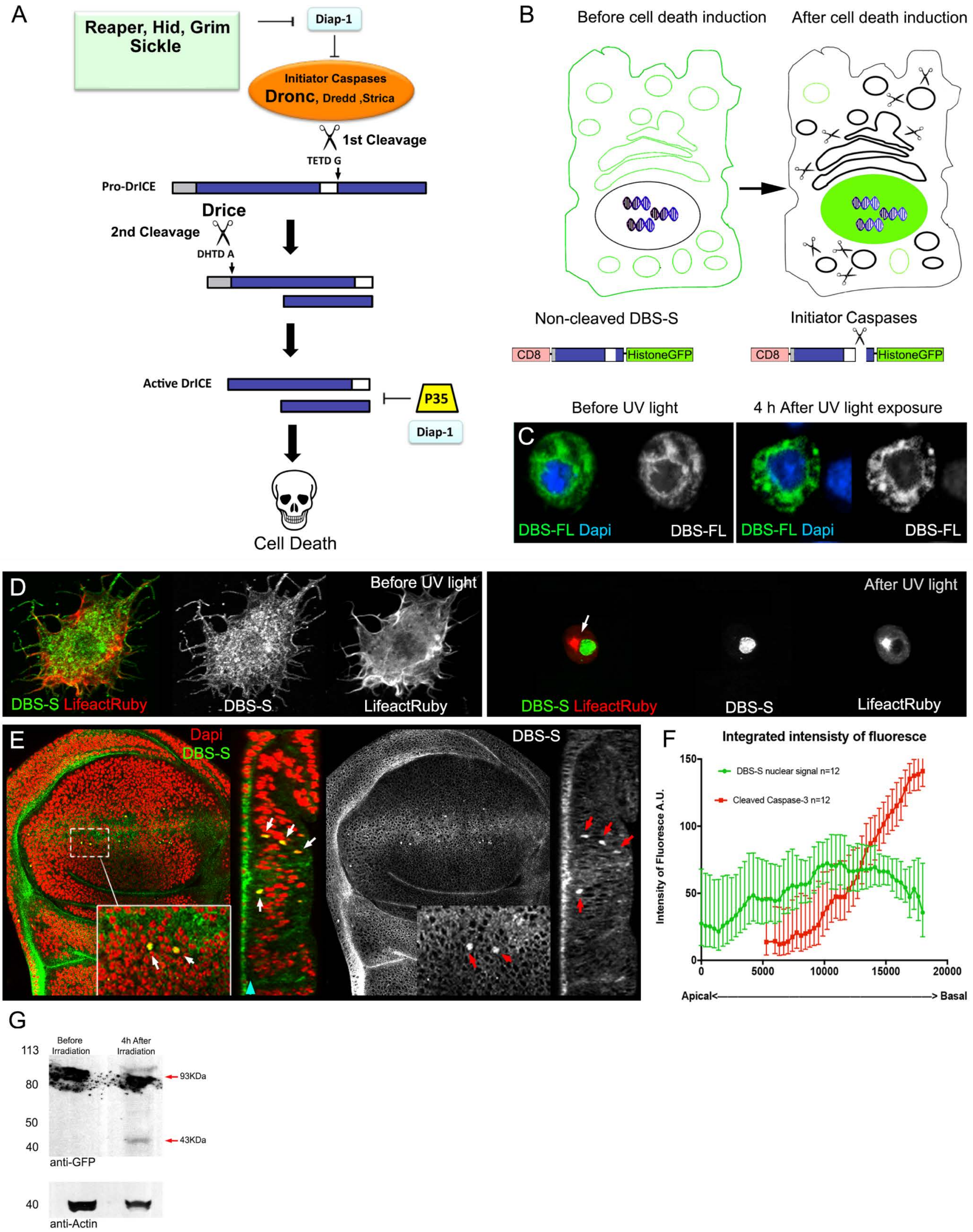

Development • Supplementary information

Figure S1. Rational design of Drice-based sensor (DBS).

A) Schematic diagram of the apoptotic program and Drice cleavage activation steps. Relevant pro-apoptotic factors (Hid, Grim, Reaper, Sickle), apical caspases (Dronc, Dredd and Strica) and apoptosis inhibitors (Diap-1 and P35) are also indicated. Notice that the first cleavage event of Drice activation is mainly mediated by Dronc, while the second demands the participation of active Drice. **B**) Diagram illustrating the predicted behaviour of DBS-S with and without apoptotic stimuli. The histone-GFP fragment should be retained at the membranes before cell death induction, whereas it should be translocated into the nucleus upon apical caspase cleavage. **C**) Behaviour of DBS-full length construct in S2 cells (DBS-FL > green, Dapi stains the nuclei). Notice DBS-FL remains attached to the membrane 4 hours after UV light treatment. **D**) S2-R+ cells grown on poly-lysine coverslips, before and after UV light treatment. DBS-S sensor is shown in green while LifeAct-Ruby labels actin in red. Both proteins are expressed under the regulation of the *actin* promoter. A multicistronic plasmid produces both proteins from a unique open reading frame (*actin* mitoPlum-2A-lifeAct-Ruby-2A-DBS-S). Notice the DBS-S translocation into the nuclei after UV light treatment, as well as contracted actin around the nucleus (white arrow). **E**) Performance of DBS-short (green) in a wild-type disc (Dapi stains nuclei in false red colour). Panels in E show an XY image of a wild-type wing disc, a magnification inset and a cross-section of the wing epithelium (Z-section). White and red arrows indicate cells positively labelled with DBS-S; notice the nuclear translocation of the histone-GFP and the expected co-localization with Dapi. Blue and red arrowheads indicate apical and basal regions of the epithelium, respectively. Note labelled nuclei with DBS-S (white arrows) are not fragmented during the delamination towards the basal surface of the epithelium. Please see MM to obtain full description of the experimental genotypes displayed in each figure. **F)** Average intensity of fluorescence (A.U. arbitrary units) of nuclear DBS-S signal (green) and cleaved caspase-3 (red) along the apico-basal axis. Notice that the GFP signal rapidly increases in apical positions while the strong cleaved caspase-3 staining is only detected from the medial to the basal end of the epithelium (n=15, error bars indicate standard deviation). **G)** Western Blot showing the specific cleavage of DBS upon irradiation; the full-length size of DBS-S is 93KDa while the processed form is 43KDa. Notice the appearance of the 43 KDa band upon irradiation as well as the absence of unspecific cleavage products.

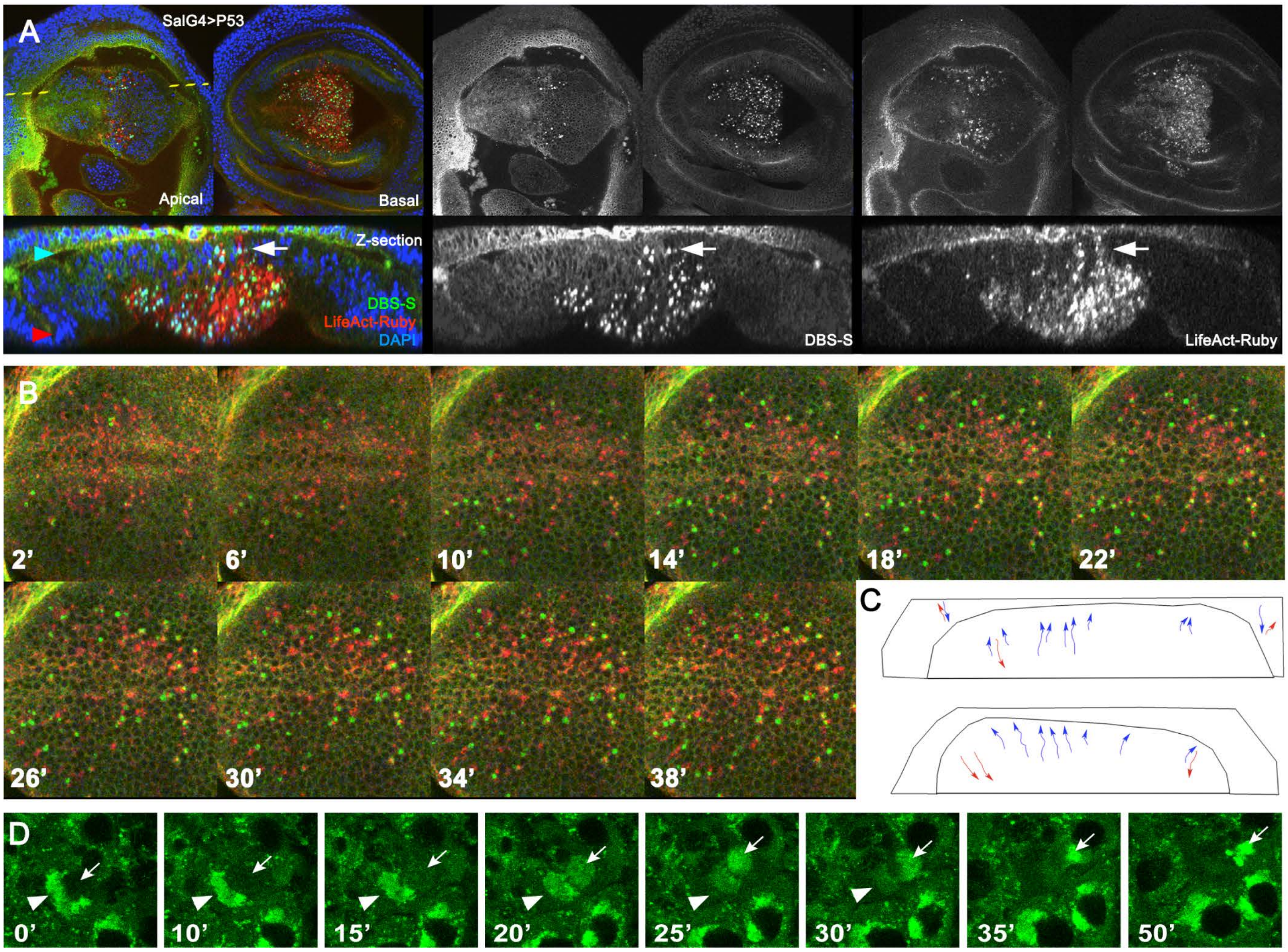

Figure S2. Labelling of caspase-activating cells through DBS-S

A) The overexpression of P53 under the control of *spalt*-Gal4 induces prominent cell death. XY images at different focal planes and cross-sections of the wing discs (Z-section) are shown in A. The discontinuous yellow line indicates the location of the Z-section in the XY image. Caspase-activating cells are positively labelled by the nuclear translocation of a *histone*-GFP fragment from DBS-S (green); actin is labelled with LifeAct-Ruby in red; nuclei are stained with Dapi. **B**) Time-lapse of a wing imaginal disc *ex vivo* after irradiation (apical view of the wing discs; time is indicated in minutes; DBS-S is show in green; LifeAct-Ruby shows actin in red). Notice the progressive accumulation of GFP positive nuclei in apical positions of the wing epithelia. The recording started 90 minutes after irradiation and it lasted for 40 minutes. Images were acquired with a 2 minutes interval. **C**) Nuclei trajectories observed in time-lapses from irradiated discs (real information shown in movie 3 and movie 4). Blue arrows indicate apical interkinetic nuclear movement, while red arrows highlight the nuclear trajectory of delaminating cells.

D) Time-lapse series of an abdominal larval epithelial cell undergoing apoptosis during metamorphosis (white arrow, DBS-S > green). Notice that the GFP signal is initially located in the membrane bound cytoplasmic structures (arrowhead points ER/Golgi) and progressively translocates to the nucleus (arrow). Strong nuclear GFP signal is only distinguishable 20 minutes after movie starts, when presumably high levels of caspase activation are achieved. Nuclear GFP accumulation is correlated with the concomitant depletion of the signal from the cytoplasm. Time is indicated in minutes in all figure panels.

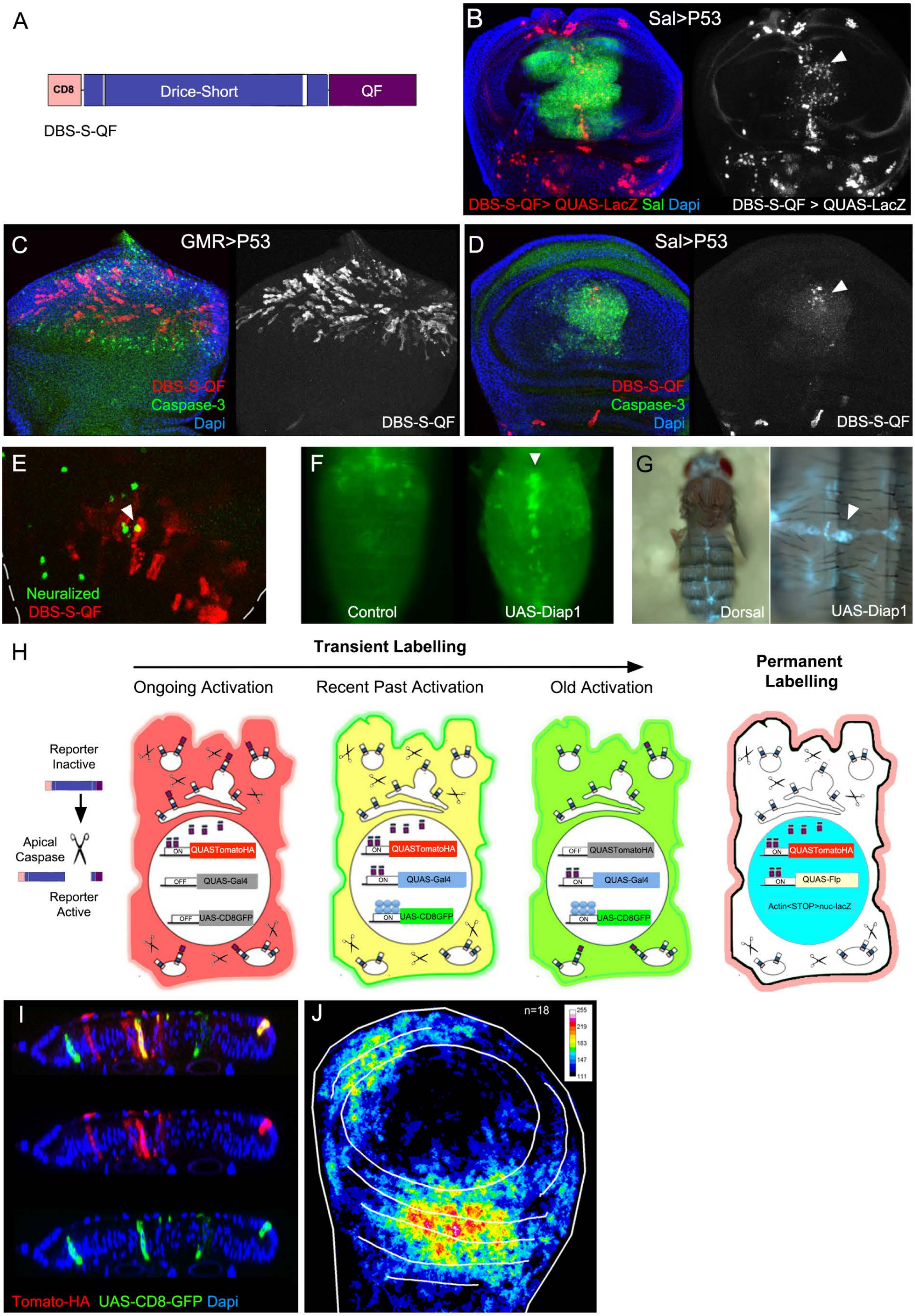

Development • Supplementary information

Figure S3. Design and functional characterization of DBS-S-QF.

A) Schematic diagram illustrating the rational design of DBS-S-QF. **B)** Induced cell death and DBS-S-QF activation in a wing imaginal disc expressing P53 under the regulation of *spalt*-Gal4. Nuclear beta-gal staining indicates the activation of DBS-S-QF (QUAS-nucLacZ, anti-betagal, red); UAS-CD8-GFP is shown in green; Dapi staining labels the nuclei. **C and D**) Induced cell death and DBS-S-QF activation in eye or wing imaginal discs expressing P53, under the regulation of either GMR promoter or *spalt*-Gal4, respectively. QUAS-Tomato-HA signal indicates the activation of DBS-S-QF (QUAS-tomato-HA, anti-HA, red); caspase-3 staining is shown in green; Dapi staining labels the nuclei. **E**) Transient labelling of caspase-activating cells in the wing discs obtained with DBS-S-QF (QUAS-tomato-HA, anti-HA, red); sensory organ precursors are labelled by neuralized-nuclear lac-Z staining in green). **F** and **G**) The ectopic expression of Diap-1 induced by DBS-S-QF activation compromises the apoptosis of larval cells in pupal stages (GFP cells indicated by white arrow in E), and ultimately causes dorsal closure defects in adults (abnormal accumulation of larval cells in adult abdomen is shown in green in F). To obtain a full description of genotypes, please see MM. **H**) Diagram showing the labelling system used in combination with DBS-S-QF to visualize the temporal patterns of apical caspase activation. The combination of different markers allows the labelling of caspase-activating cells transiently or permanently. The current/ongoing activation of caspases is shown in red upon induction of QUAS-Tomato-HA (anti-HA in red). Concomitantly, QF translocation can also induce the expression of a Gal4 transcription factor (QUAS-Gal4). Gal4 production can then trigger the expression of a second cellular marker (UAS-CD8-GFP). The appearance of the GFP signal is delayed in respect to the QUAS-Tomato since it demands a second round of transcriptional events; however, both markers can co-exist (yellow cells; yellow indicates either sustained caspase activation or alternatively, sensor activation in the recent past). If apical caspase activation terminates QF translocation finishes; however, the transcriptional amplification obtained with the Gal/UAS transcription loop can maintain the GFP signal for long periods of time after the QUAS-tomato signal disappears (Old caspase activation, green cells). Lineage-tracing (permanent labelling) of caspase activating cells is also achievable by using DBS-QF. In this case the QF transcription factor activates a recombinase (QUAS-flipase), which subsequently mediates the genomic excision of a FRT-stop cassette. Before recombination, the FRT-stop cassette prevents the activation of a cellular marker (nuclear-lacZ) under the regulation of an *actin* ubiquitous promoter (permanent labelling). **I**) Representative example of H. To obtain a full description of genotypes, please see MM. **J)** An accumulative plot of clones growing in the wing disc obtained through the permanent labelling of caspase-activating cells with DBS-QF (number of wing discs analysed n=18, average number of lacZ clones per disc 21.5 ± 2.64). Warmer areas (red) in the heat map indicate higher concentration of clones. Notice that clones tend to preferentially grow in the proximal regions of the wing discs (wing hinge), remaining distal parts (wing blade) less crowded.

Development • Supplementary information

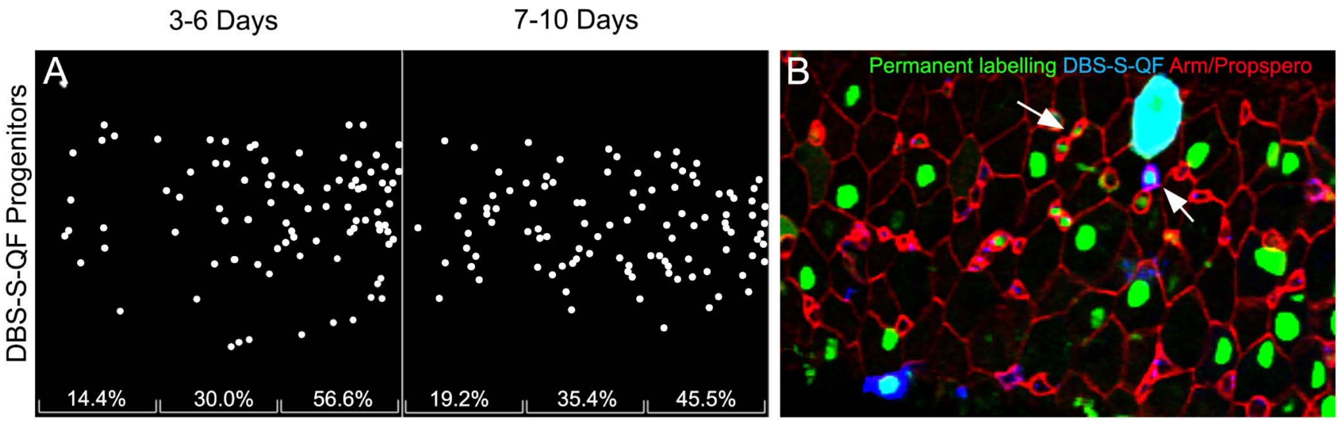

Figure S4. Transient and permanent labelling of caspase-activating cells in the posterior midgut.

A) Quantification of intestinal cell progenitors (intestinal stem cells and enteroblasts) labelled in the gut with DBS-S-QF. Brackets delimit the arbitrary subdivision made for making these quantifications; % indicates the relative number of intestinal progenitors activating the sensors respect to the total. DBS-S-QF: 3-6d n = 13, 7-10d n = 24. **B**) Representative image of an adult posterior midgut showing caspase-activating cells permanently labelled with DBS-S-QF (the historical caspase activation, nuclear lac-Z is shown in green, ongoing DBS-S-QF activation is shown in blue (anti-HA); armadillo/prospero staining in red). White arrows indicate intestinal progenitors labelled permanently with DBS-S-QF.

Development • Supplementary information

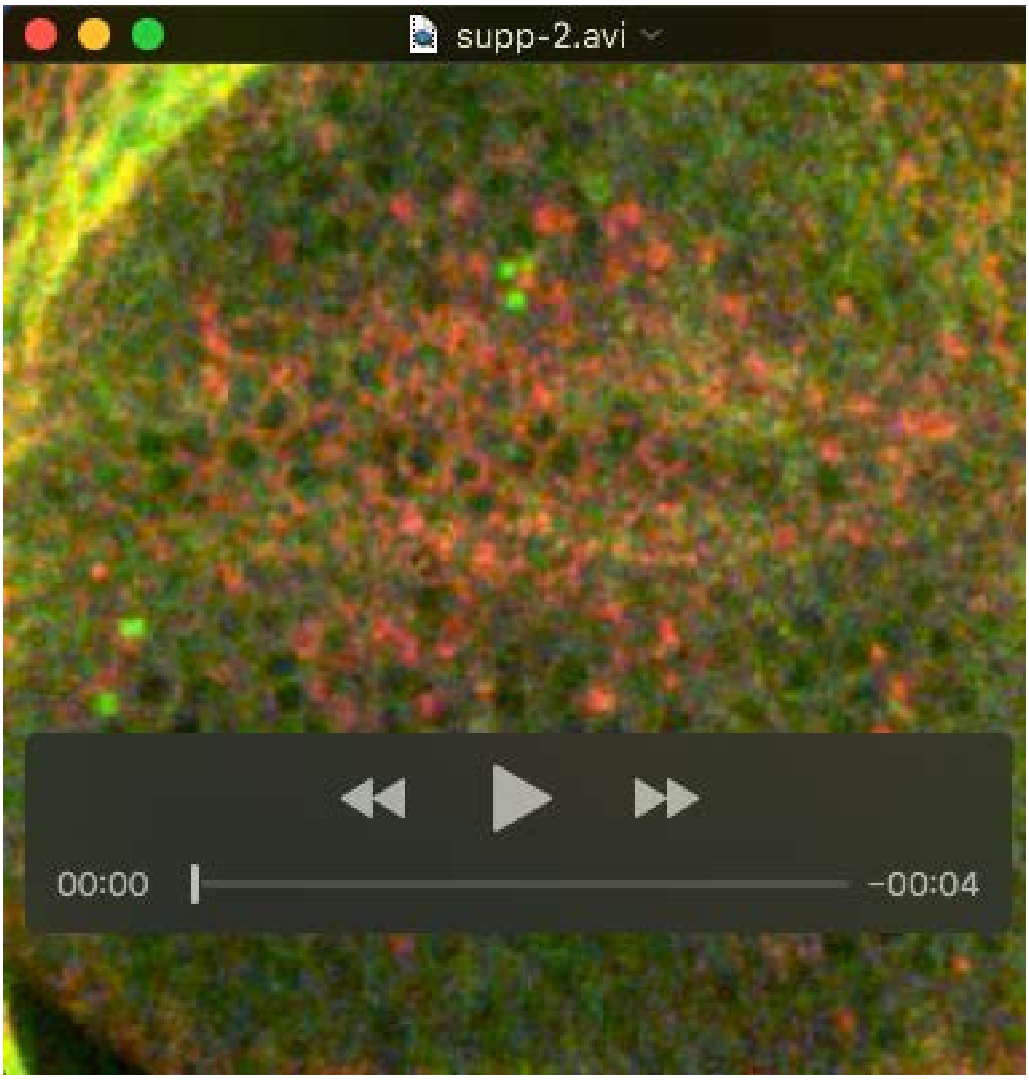

Movie 1. Time-lapse *ex vivo* of a wing imaginal disc after irradiation. Nuclei marked in green indicate DBS-S activation, LifeAct-Ruby shows actin in red. The time-lapse is showing an apical focal plane of the wing disc. Notice the progressive accumulation of GFP-positive nuclei in apical focal planes. The recording started 90 minutes after irradiation and it lasted for 40 minutes. Images were acquired within a 2 minutes interval.

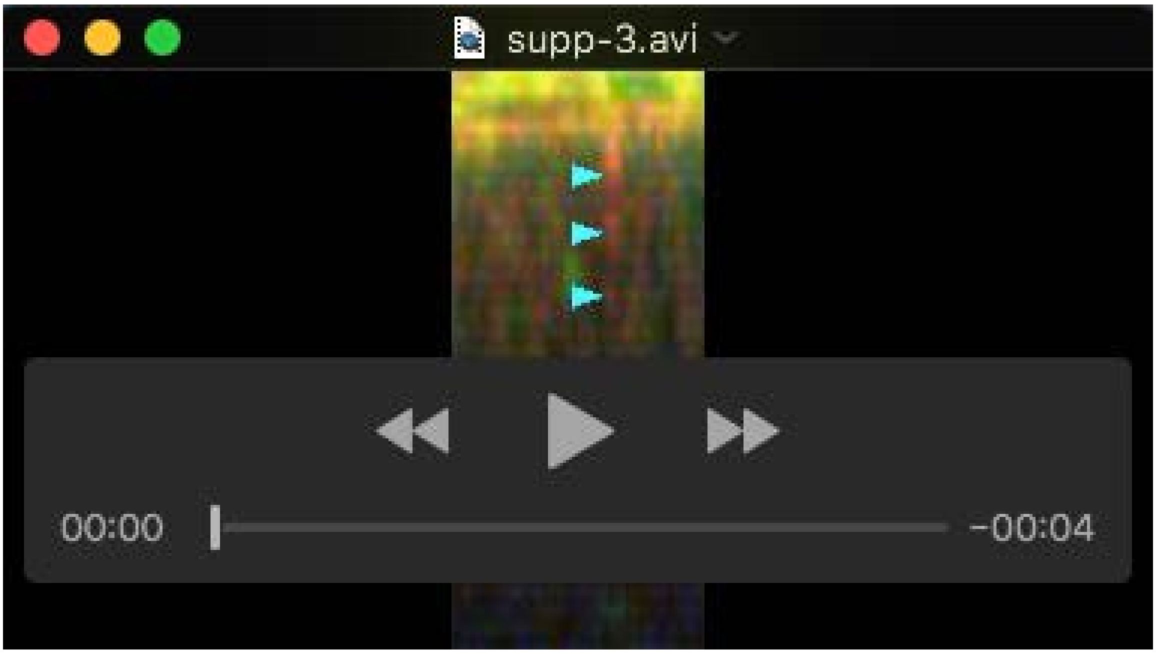

Movie 2. Detail of nuclear apical migration in apoptotic cells. Nuclei marked in green indicate DBS-S activation, LifeAct-Ruby shows actin in red. The time-lapse is showing a cross-section of a wing imaginal disc. Notice the progressive accumulation of GFP signal in the nuclei, the changes in their shape (roundness increase) and the movement towards the apical cell cortex. Notice also the contraction of the actin bundles over time (blue arrowheads). The recording started 90 minutes after irradiation and it lasted for 40 minutes. Snapshots were acquired every 2 minutes.

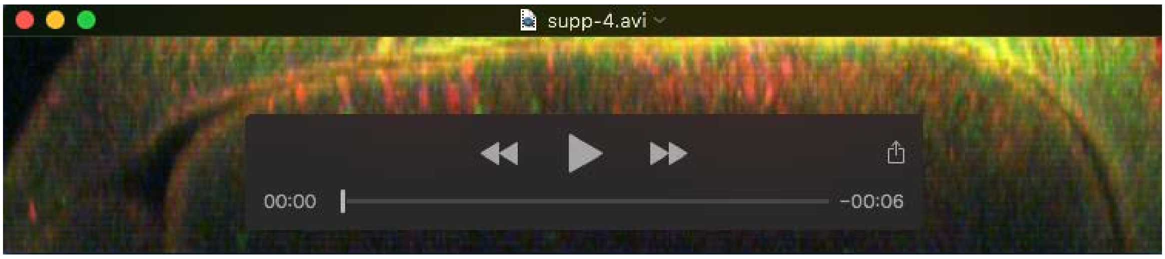

Movie 3. General view of the wing disc shown in movie 2. Nuclei marked in green indicate DBS-S activation, LifeAct-Ruby shows actin in red. The time-lapse is showing a cross-section of a wing imaginal disc. Notice the progressive accumulation of GFP signal in the nuclei, the changes in their shape (roundness increase) and the movement towards the apical cell cortex (white arrowheads). Notice also the contraction of the actin bundles over time (blue arrowheads). The recording started 90 minutes after irradiation and it lasted for 40 minutes. Snapshots were acquired every 2 minutes.

Development • Supplementary information

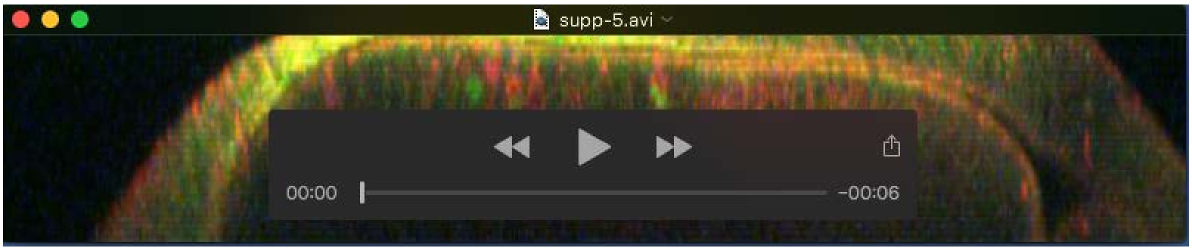

Movie 4. General view of the wing disc showing the delamination of apoptotic cells
Nuclei marked in green indicate DBS-S activation, LifeAct-Ruby shows actin in red. The time-lapse is showing a cross-section of a wing imaginal disc. Notice the progressive the migration of GFP positive nuclei together with actin-enriched structures towards the basal side of the wing epithelium (white arrowheads). The recording started 90 minutes after irradiation and it lasted for 40 minutes. Snapshots were acquired every 2 minutes.

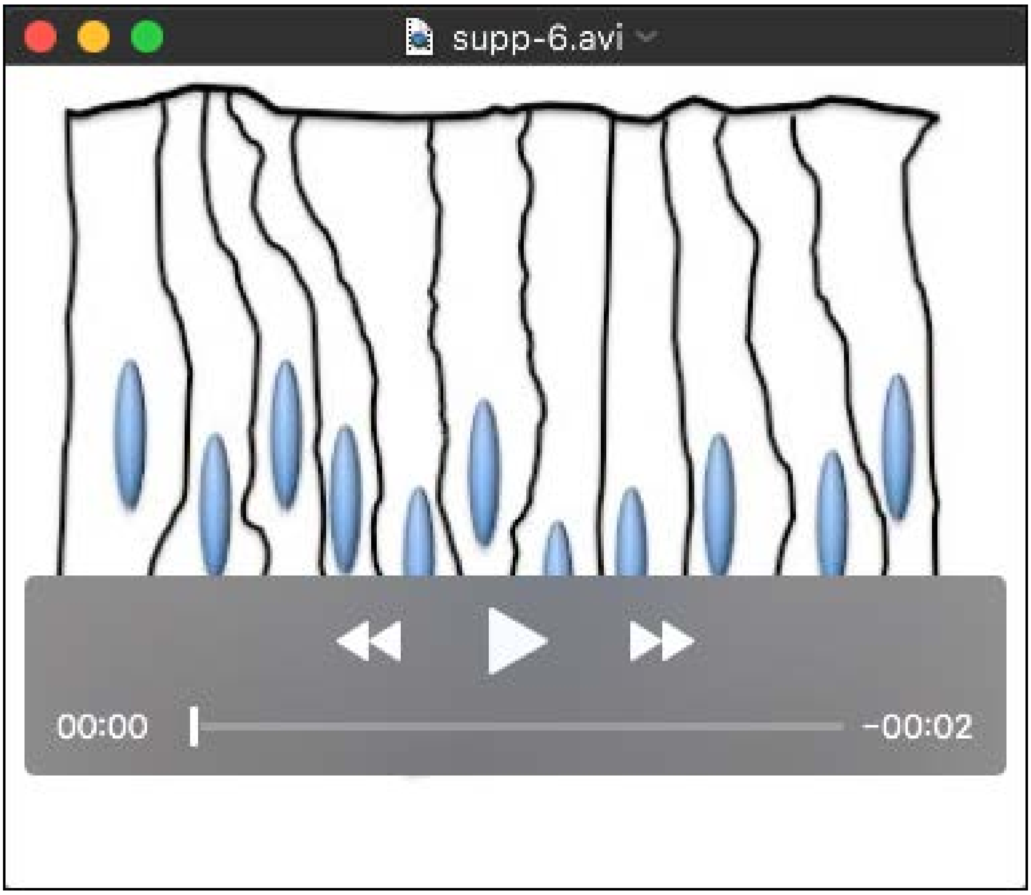

Movie 5. Schematic diagram summarizing the apoptotic events uncovered with DBS-S. DBS-S translocation into the nucleus is represented in green. Changes in actin dynamics are illustrated in red.

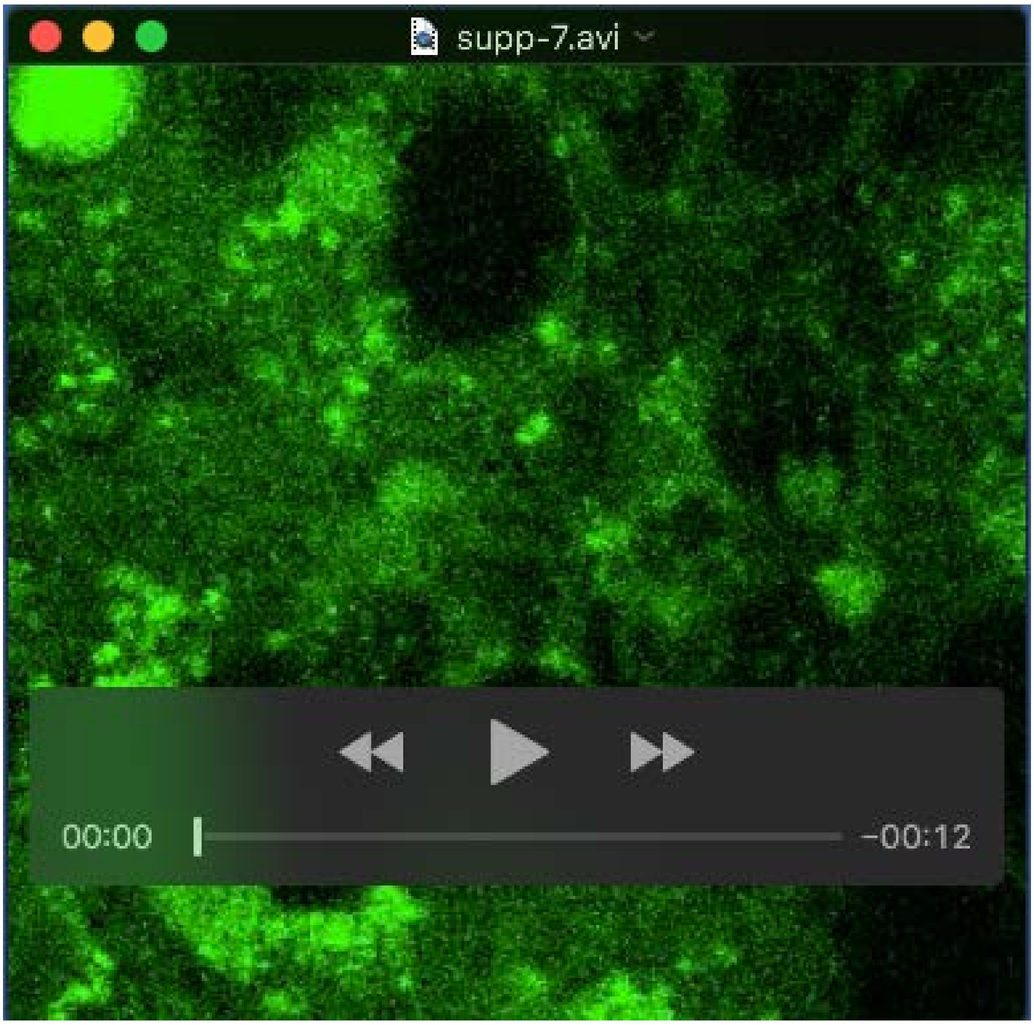

Movie 6. Tracking of apoptotic larval epithelial cells (LECs) in the fly abdomen cells during pupal stages with DBS-S. In non-dying cells the GFP signal from DBS-S is retained at the cellular membranes (arrowhead point ER-Glogi structures), remaining the nuclei unlabelled. Arrow highlights a dying LEC that progressively accumulates the GFP signal into the nucleus. Strong nuclear GFP signal is only distinguishable 20 minutes after movie starts, when presumably high levels of caspase activation are achieved. Nuclear accumulation of GFP is correlated with the concomitant depletion of the signal from the cytoplasm. At the end of the movie, the nucleus of the apoptotic LEC is fragmented. A region of a hemi-segment of segment A2 is shown. The time-length of the movie was 2.6 hours and frames were acquired every 150 seconds. Anterior is to the left. Dorsal is up.

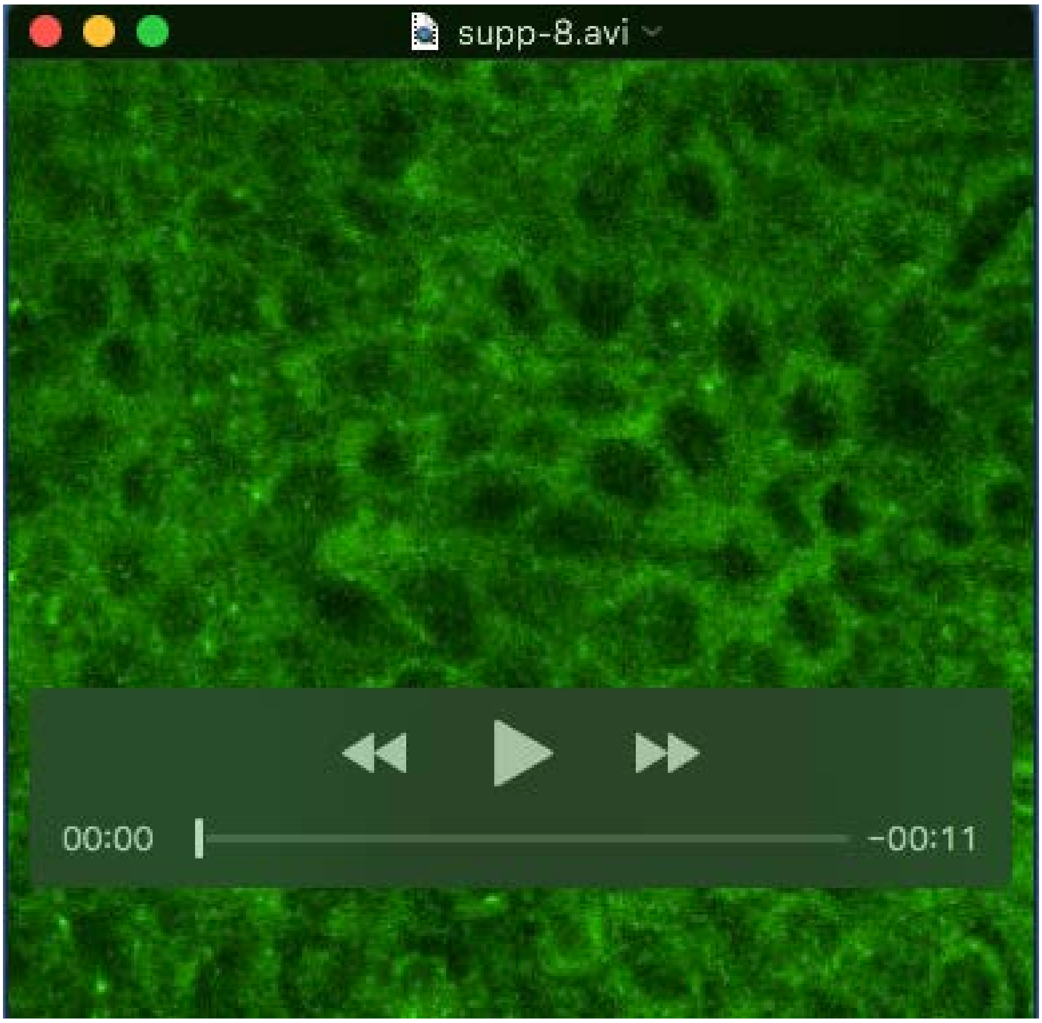

Movie 7. Tracking of apoptotic histoblasts in the fly abdomen during pupal stages with DBS-S. In non-dying histoblasts the GFP signal from DBS-S is retained at the cellular membranes and the nuclei remain unlabelled. Arrows highlight dying histoblasts that progressively accumulates the GFP signal into the nucleus. Eventually the nucleus of the apoptotic cells is fragmented and the dying/dead cell delaminates. A region of a hemi-segment of segment A2 is shown. The time-length of the movie was 52.5 minutes and frames were acquired every 150 seconds. Anterior is to the left. Dorsal is up.

Development • Supplementary information

Table S1. Full description of experimental genotypes

Experimental Genotypes
w,Tubulin-DBS-S; UAS-hid (BL65403)/*spalt*^{EPV}-Gal4 (Fig. 1C, Fig. S1F)
w; UAS-Hep^{Act} (9306), Tubulin-DBS-S /*spalt*^{EPV}-Gal4 (Fig. 1D)
yw heat shock-flipase/Tubulin-DBS-S; FRT40 w+/FRT40 *Minute* arm-LacZ (Fig. 1E)
w;;Actin mitoPlum-2A-lifeAct-Ruby-2A-DBS-S (Fig. 2, Fig. S12B, Movies 1-4)
w;Tubulin-DBS-S/Cyo (Fig. S1E)
w; UAS-Hep.Act (9306), Tubulin-DBS-S /*spalt*EPV-Gal4; *Dronc*^{L29} (Fig. 3A,B)
w; UAS-Hep.Act, Tubulin-DBS-S /TubG80ts (from BL7019); *hedgehog*-Gal4/UAS-P35 (BL5073) (Fig. 3C)
w; UAS-Hid, Tubulin-DBS-S /TubG80ts (BL7019); *hedgehog*-Gal4/UAS-P35 (Fig. 3D)
Actin DBS-S-QF, UAS-mCD8-GFP(from BL30118), QUAS-tomato-HA (from BL30118)/+ (Fig. 4A)
Actin DBS-S-QF, UAS-mCD8-GFP (from BL30118), QUAS-tomato-HA/+; GMR-Hid (from BL BL5251) (Fig. 4B)
Actin DBS-S-QF, UAS-mCD8-GFP, QUAS-tomato-HA; *ap*^{md544}-Gal4 /+ (Fig. 4C)
Actin DBS-S-QF, UAS-mCD8-GFP, QUAS-tomato-HA; *ap*^{md544}-Gal4 /UAS-*Dronc*-RNAi (gift from Pascal Meier, Institute of Cancer Research, London) (Fig. 4D)
Actin DBS-S-QF, UAS-mCD8-GFP, QUAS-tomato-HA/+; *spalt*^{EPV}-Gal4/GUSDp53 (BL6584) (Fig. 4E)
Actin DBS-S-QF, UAS-mCD8-GFP, QUAS-tomato-HA/+; *spalt*^{EPV}-Gal4/GUS-Dp53; UAS-P35/+ (Fig. 4F)
Actin DBS-S-QF, UAS-mCD8-GFP, QUAS-tomato-HA/+;; QUAS-Gal4 (Fig. 4G, Fig. S3I and S3G)
Actin DBS-S-QF, UAS-mCD8-GFP, QUAS-tomato-HA/+; QUAS-FLPo (BL30126) /+; Actin5C FRT-stop-FRT lacZ-nls/+ (BL6355) (Fig. 4H, Figs S3J and S4B)

Actin DBS-S-QF, UAS-mCD8-GFP, QUAS-tomato-HA/+;; (Fig. 5, Fig. S4A)
w; GUS-P53/*spalt*^{EPV}-Gal4; Actin mitoPlum-2A-lifeAct-Ruby-2A-DBS-S/+ (Fig. S2A)
Actin DBS-S-QF, UAS-mCD8-GFP, QUAS-nucLacZ.7 (BL30006)/+; *spalt*^{EPV}-Gal4/GUS-Dp53 (BL6584) (Fig. S3B)
Actin DBS-S-QF, UAS-mCD8-GFP, QUAS-tomato-HA/+;;GMR-Dp53 (BL8417) (Fig. S3C)
w, Actin DBS-S-QF, UAS-mCD8-GFP, QUAS-tomato-HA/+; GUS-P53/*spalt*^{EPV}-Gal4 (Fig. S3D)
Actin DBS-S-QF, UAS-mCD8-GFP, QUAS-tomato-HA/+; *neur*^{A101} (BL4369)/+ (Fig. S3E)
Actin DBS-S-QF, UAS-mCD8-GFP, QUAS-tomato-HA/+;; QUAS-Gal4/UAS-Diap1 (Fig. S3F,G)

Development • Supplementary information
